# (μ-Ethane-1,2-diamine-κ^2^ *N*:*N*′)bis­[bis­(ethane-1,2-diamine-κ^2^ *N*,*N*′)zinc(II)] tetra­kis­(perchlorate)

**DOI:** 10.1107/S1600536810038730

**Published:** 2010-10-02

**Authors:** Man-Hua Ding, Seik Weng Ng

**Affiliations:** a Department of Biology and Chemistry, Hunan University of Science and Engineering, Yongzhou, Hunan 425100, People’s Republic of China; b Department of Chemistry, University of Malaya, 50603 Kuala Lumpur, Malaysia

## Abstract

In the title salt, [Zn_2_(C_2_H_8_N_2_)_5_](ClO_4_)_4_, an ethyl­enediamine mol­ecule bridges two bis­(ethyl­enediamine)­zinc units; the five-coordinate Zn atoms show a trigonal–bipyramidal coordination geometry that is distorted towards square-pyramidal (that of one Zn atom is distorted by 12% and that of the other by 34%). The perchlorate ions are all disordered over two positions in a 1:1 ratio. The cation inter­acts weakly with the anion by N—H⋯O hydrogen bonds, generating a three-dimensional network.

## Related literature

For other μ-(ethyl­enediamine)­bis­[bis­(ethyl­enediamine)­zinc(II)] salts, see: Khan *et al.* (2003 ▶); Natarajan *et al.* (2006 ▶); Qi *et al.* (2007 ▶).
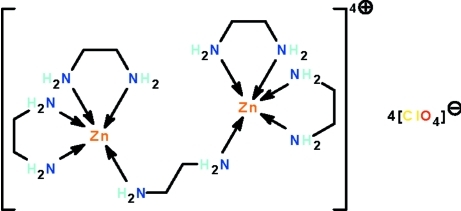


## Experimental

### 

#### Crystal data

[Zn_2_(C_2_H_8_N_2_)_5_](ClO_4_)_4_
*M* *_r_* = 829.06
Monoclinic, 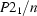

*a* = 15.6297 (8) Å
*b* = 14.3133 (7) Å
*c* = 15.6811 (8) Å
β = 119.636 (1)°
*V* = 3049.1 (3) Å^3^
*Z* = 4
Mo *K*α radiation
μ = 2.01 mm^−1^
*T* = 248 K
0.45 × 0.40 × 0.10 mm

#### Data collection

Bruker SMART APEX diffractometer
Absorption correction: multi-scan (*SADABS*; Sheldrick, 1996 ▶) *T*_min_ = 0.711, *T*_max_ = 1.000
14146 measured reflections
6543 independent reflections
4259 reflections with *I* > 2σ(*I*)
*R*_int_ = 0.029

#### Refinement

*R*[*F* ^2^ > 2σ(*F* ^2^)] = 0.056
*wR*(*F* ^2^) = 0.186
*S* = 1.05
6543 reflections
535 parameters
293 restraints
H-atom parameters constrained
Δρ_max_ = 1.06 e Å^−3^
Δρ_min_ = −0.83 e Å^−3^

### 

Data collection: *SMART* (Bruker, 2003 ▶); cell refinement: *SAINT* (Bruker, 2003 ▶); data reduction: *SAINT*; program(s) used to solve structure: *SHELXS97* (Sheldrick, 2008 ▶); program(s) used to refine structure: *SHELXL97* (Sheldrick, 2008 ▶); molecular graphics: *X-SEED* (Barbour, 2001 ▶); software used to prepare material for publication: *publCIF* (Westrip, 2010 ▶).

## Supplementary Material

Crystal structure: contains datablocks global, I. DOI: 10.1107/S1600536810038730/hg2718sup1.cif

Structure factors: contains datablocks I. DOI: 10.1107/S1600536810038730/hg2718Isup2.hkl

Additional supplementary materials:  crystallographic information; 3D view; checkCIF report

## Figures and Tables

**Table 1:** Hydrogen-bond geometry (Å, °)

*D*—H⋯*A*	*D*—H	H⋯*A*	*D*⋯*A*	*D*—H⋯*A*
N1—H12⋯O1	0.86	1.93	2.790 (9)	174
N1—H13⋯O8′	0.86	2.22	3.066 (12)	169
N3—H32⋯O12	0.86	2.34	3.018 (7)	136
N3—H32⋯O12′	0.86	2.29	3.070 (10)	152
N4—H41⋯O7^i^	0.86	2.35	3.157 (12)	156
N4—H41⋯O7′^i^	0.86	2.08	2.885 (9)	156
N4—H42⋯O16′	0.86	2.12	2.973 (8)	175
N5—H51⋯O1	0.86	2.30	3.120 (15)	159
N6—H62⋯O2	0.86	2.29	3.034 (11)	146
N7—H71⋯O2	0.86	2.32	3.163 (10)	168
N7—H71⋯O2′	0.86	2.22	3.018 (10)	154
N7—H74⋯O6^ii^	0.86	2.35	3.143 (11)	154
N7—H74⋯O6′^ii^	0.86	2.36	3.210 (9)	172
N8—H81⋯O11^ii^	0.86	2.26	3.075 (8)	157
N8—H81⋯O11′^ii^	0.86	2.29	3.148 (12)	177
N10—H102⋯O3^i^	0.86	2.17	2.929 (9)	146

Symmetry codes: (i) 
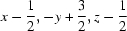
; (ii) 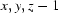.

**Fig. 1.**
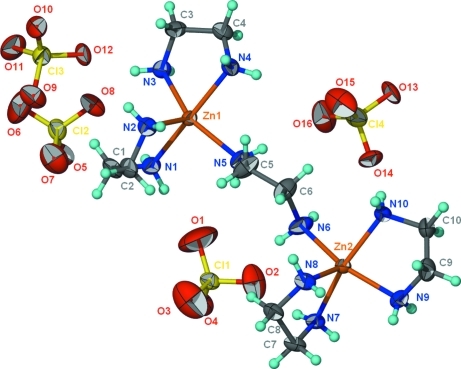
Thermal ellipsoid plot (Barbour, 2001) of [Zn2(C2H8N2)5] 4[ClO4] the 50% probability level; hydrogen atoms are shown as spheres of arbitrary radius. The disorder in the ethylenediamine perchlorate parts is not shown.

## supplementary crystallographic information

### Comment

The zinc(II) cation furnishes a number of compounds with ethylenediamine, and there are serveral salts having the atom chelated by either two or three of the ligands. There is a much smaller number of compounds having the ligand behaving in a bridging mode, and the title zinc perchlorate complex (Scheme I) is an unusual example of a 1:1.5 adduct. µ-(Ethane-1,2-diamine)-bis[bis(ethane-1,2-diamine)zinc(II)] tetrakisperchlorate (Fig. 1) which is a dinuclear compound with the metal atoms in a five-coordinate environment. The geometries are both trigonal bipyramidal,but one is distorted towards a square pyramid 12% and whereas the other is distorted by 34% (along the Berry pseudorotation pathway). The perchlorate anions are only weakly linked to the dinuclear unit by hydrogen bonds; the anions are all disordered in their oxygen atoms.

Other µ-(ethylenediamine)-bis[bis(ethylenediamine)zinc(II)] tetracations have been reported, but the counterions are the extremely large polyoxometallate counterions (Khan *et al.*, 2003; Natarajan *et al.*, 2006; Qi *et al.*, 2007).

### Experimental

A methanol solution (10 ml) of diaminoethane (1.20 g, 0.02 mol) was added to a methanol solution (50 ml) of zinc acetate (2.48 g, 0.01 mol). The mixture was filtered, and to the solution was added an aqueous solution of sodium perchlorate (2.44 g, 0.02 mol). After several days, colorless crystals were separated from solution.

### Refinement

Carbon-bound and nitrogen H-atoms were placed in calculated positions (C–H 0.98 Å, N–H 0.86 Å) and were included in the refinement in the riding model approximation, with *U*(H) set tied as 1.2*U*(C).

The ethyl portion of two ethylenediamine units are disordered over two positions; the N–C distances in the disordered units were tightly restrained to 1.470±0.005 Å and the C–C distances to 1.540±0.005 Å.

The perchlorate ions are all disordered over two positions; as the disorder refined to a nearly 1:1 ratio, the ratio was fixed as exactly 1:1. The Cl–O distance was tightly restrained to 1.410±0.005 Å and the O···O distance to 2.30±0.010 Å; the anisotropic temperature factors were restrained to be nearly isotropic.

Some 293 restraints are necessary to stabilize the refinement. The final difference Fourier map had a peak in the vicinity of O11.

### Crystal data

**Table:**

[Zn_2_(C_2_H_8_N_2_)_5_](ClO_4_)_4_	*F*(000) = 1704
*M**_r_* = 829.06	*D*_x_ = 1.806 Mg m^−^^3^
Monoclinic, *P*2_1_/*n*	Mo *K*α radiation, λ = 0.71073 Å
Hall symbol: -P 2yn	Cell parameters from 4985 reflections
*a* = 15.6297 (8) Å	θ = 2.6–26.9°
*b* = 14.3133 (7) Å	µ = 2.01 mm^−^^1^
*c* = 15.6811 (8) Å	*T* = 248 K
β = 119.636 (1)°	Prism, colorless
*V* = 3049.1 (3) Å^3^	0.45 × 0.40 × 0.10 mm
*Z* = 4	

### Data collection

**Table:**

Bruker SMART APEX diffractometer	6543 independent reflections
Radiation source: fine-focus sealed tube	4259 reflections with *I* > 2σ(*I*)
graphite	*R*_int_ = 0.029
φ and ω scans	θ_max_ = 27.1°, θ_min_ = 2.1°
Absorption correction: multi-scan (*SADABS*; Sheldrick, 1996)	*h* = −19→17
*T*_min_ = 0.711, *T*_max_ = 1.000	*k* = −18→17
14146 measured reflections	*l* = −20→15

### Refinement

**Table:**

Refinement on *F*^2^	Primary atom site location: structure-invariant direct methods
Least-squares matrix: full	Secondary atom site location: difference Fourier map
*R*[*F*^2^ > 2σ(*F*^2^)] = 0.056	Hydrogen site location: inferred from neighbouring sites
*wR*(*F*^2^) = 0.186	H-atom parameters constrained
*S* = 1.05	*w* = 1/[σ^2^(*F*_o_^2^) + (0.1053*P*)^2^ + 3.3033*P*] where *P* = (*F*_o_^2^ + 2*F*_c_^2^)/3
6543 reflections	(Δ/σ)_max_ = 0.001
535 parameters	Δρ_max_ = 1.06 e Å^−^^3^
293 restraints	Δρ_min_ = −0.83 e Å^−^^3^

### Fractional atomic coordinates and isotropic or equivalent isotropic displacement parameters (Å^2^)

**Table:**

	*x*	*y*	*z*	*U*_iso_*/*U*_eq_	Occ. (<1)
Zn1	0.30084 (4)	0.63583 (4)	0.51082 (4)	0.03254 (19)	
Zn2	0.33807 (4)	0.63741 (4)	0.06722 (4)	0.0331 (2)	
Cl1	0.60325 (9)	0.61482 (11)	0.44862 (11)	0.0460 (4)	
Cl2	0.56091 (10)	0.60846 (13)	0.89575 (12)	0.0568 (4)	
Cl3	0.31237 (11)	0.85357 (11)	0.78206 (11)	0.0503 (4)	
Cl4	0.12173 (11)	0.43344 (11)	0.18233 (13)	0.0558 (4)	
O1	0.5436 (10)	0.6079 (13)	0.4913 (11)	0.166 (8)	0.50
O2	0.5520 (8)	0.5753 (7)	0.3528 (5)	0.096 (4)	0.50
O3	0.6263 (10)	0.7073 (5)	0.4396 (12)	0.134 (6)	0.50
O4	0.6906 (7)	0.5627 (9)	0.5042 (10)	0.064 (5)	0.50
O5	0.6386 (7)	0.5439 (7)	0.9279 (10)	0.067 (4)	0.50
O6	0.5554 (11)	0.6492 (11)	0.9740 (8)	0.147 (7)	0.50
O7	0.5853 (8)	0.6838 (6)	0.8500 (9)	0.101 (4)	0.50
O8	0.4708 (6)	0.5706 (8)	0.8249 (7)	0.058 (4)	0.50
O9	0.3829 (8)	0.9031 (9)	0.7681 (10)	0.072 (5)	0.50
O10	0.2323 (5)	0.9136 (6)	0.7589 (8)	0.073 (3)	0.50
O11	0.3578 (8)	0.8256 (8)	0.8813 (4)	0.083 (3)	0.50
O12	0.2798 (7)	0.7759 (6)	0.7193 (7)	0.059 (3)	0.50
O13	0.0504 (7)	0.3625 (6)	0.1359 (8)	0.061 (3)	0.50
O14	0.1800 (7)	0.4358 (7)	0.1363 (7)	0.070 (3)	0.50
O15	0.0743 (7)	0.5201 (5)	0.1690 (11)	0.102 (4)	0.50
O16	0.1829 (8)	0.4167 (9)	0.2828 (4)	0.102 (4)	0.50
O1'	0.6889 (7)	0.5608 (9)	0.4831 (11)	0.066 (5)	0.50
O2'	0.5760 (11)	0.6527 (11)	0.3559 (6)	0.131 (5)	0.50
O3'	0.5263 (6)	0.5629 (6)	0.4472 (10)	0.087 (4)	0.50
O4'	0.6187 (8)	0.6913 (6)	0.5127 (7)	0.091 (4)	0.50
O5'	0.6431 (7)	0.5608 (8)	0.9030 (10)	0.068 (4)	0.50
O6'	0.5437 (8)	0.5684 (7)	0.9710 (6)	0.091 (4)	0.50
O7'	0.5763 (9)	0.7031 (4)	0.9143 (10)	0.111 (5)	0.50
O8'	0.4770 (8)	0.5879 (10)	0.8053 (6)	0.077 (5)	0.50
O9'	0.3966 (7)	0.9009 (10)	0.7969 (11)	0.079 (5)	0.50
O10'	0.2289 (8)	0.8949 (12)	0.7013 (10)	0.205 (10)	0.50
O11'	0.2965 (13)	0.8605 (11)	0.8627 (9)	0.133 (6)	0.50
O12'	0.3136 (12)	0.7583 (5)	0.7605 (12)	0.123 (7)	0.50
O13'	0.0736 (10)	0.3482 (6)	0.1748 (12)	0.121 (7)	0.50
O14'	0.2207 (5)	0.4184 (11)	0.2095 (14)	0.153 (7)	0.50
O15'	0.0743 (12)	0.4832 (12)	0.0934 (8)	0.187 (8)	0.50
O16'	0.1178 (12)	0.4898 (11)	0.2541 (11)	0.132 (5)	0.50
N1	0.4573 (3)	0.6420 (3)	0.6072 (4)	0.0443 (12)	
H11	0.4756	0.5997	0.6518	0.053*	0.50
H12	0.4877	0.6332	0.5748	0.053*	0.50
H13	0.4718	0.6273	0.6660	0.053*	0.50
H14	0.4868	0.6040	0.5879	0.053*	0.50
N2	0.3132 (3)	0.7821 (3)	0.5284 (4)	0.0449 (11)	
H21	0.2768	0.8101	0.4736	0.054*	0.50
H22	0.2970	0.8004	0.5707	0.054*	0.50
H23	0.2933	0.8076	0.4720	0.054*	0.50
H24	0.2752	0.8008	0.5500	0.054*	0.50
N3	0.2715 (3)	0.5914 (3)	0.6223 (3)	0.0409 (11)	
H31	0.2914	0.5348	0.6389	0.049*	
H32	0.3022	0.6264	0.6731	0.049*	
N4	0.1433 (3)	0.6078 (4)	0.4212 (3)	0.0460 (12)	
H41	0.1113	0.6595	0.4016	0.055*	
H42	0.1321	0.5754	0.3705	0.055*	
N5	0.3141 (4)	0.6054 (4)	0.3861 (3)	0.0468 (12)	
H51	0.3748	0.5941	0.4041	0.056*	
H52	0.2805	0.5562	0.3578	0.056*	
N6	0.3390 (4)	0.6155 (4)	0.2026 (4)	0.0585 (15)	
H61	0.3295	0.5571	0.2075	0.070*	
H62	0.3966	0.6294	0.2500	0.070*	
N7	0.4950 (3)	0.6368 (3)	0.1374 (3)	0.0377 (10)	
H71	0.5199	0.6217	0.1982	0.045*	0.50
H72	0.5146	0.5971	0.1095	0.045*	0.50
H73	0.5194	0.5987	0.1862	0.045*	0.50
H74	0.5142	0.6202	0.0968	0.045*	0.50
N8	0.3566 (3)	0.7837 (3)	0.0730 (3)	0.0438 (11)	
H81	0.3424	0.8051	0.0163	0.053*	0.50
H82	0.3189	0.8102	0.0913	0.053*	0.50
H83	0.3149	0.8081	0.0178	0.053*	0.50
H84	0.3464	0.8068	0.1179	0.053*	0.50
N9	0.3228 (3)	0.6115 (4)	−0.0745 (3)	0.0506 (13)	
H91	0.3526	0.6543	−0.0884	0.061*	
H92	0.3486	0.5585	−0.0746	0.061*	
N10	0.1871 (3)	0.5977 (4)	−0.0105 (3)	0.0461 (12)	
H101	0.1754	0.5579	0.0235	0.055*	
H102	0.1507	0.6459	−0.0200	0.055*	
C1	0.4788 (10)	0.7355 (6)	0.6515 (10)	0.058 (3)	0.50
H1A	0.5492	0.7498	0.6821	0.070*	0.50
H1B	0.4580	0.7412	0.7009	0.070*	0.50
C2	0.4179 (5)	0.7996 (11)	0.5634 (10)	0.055 (4)	0.50
H2A	0.4337	0.8652	0.5830	0.066*	0.50
H2B	0.4331	0.7867	0.5110	0.066*	0.50
C3	0.1651 (3)	0.5971 (5)	0.5848 (4)	0.0490 (15)	
H3A	0.1455	0.6624	0.5831	0.059*	
H3B	0.1477	0.5622	0.6278	0.059*	
C4	0.1117 (4)	0.5559 (4)	0.4816 (3)	0.0460 (14)	
H4A	0.1282	0.4895	0.4836	0.055*	
H4B	0.0404	0.5615	0.4539	0.055*	
C5	0.2795 (6)	0.6828 (4)	0.3168 (4)	0.0618 (19)	
H5A	0.3255	0.7348	0.3474	0.074*	
H5B	0.2159	0.7030	0.3087	0.074*	
C6	0.2663 (5)	0.6687 (5)	0.2151 (4)	0.0585 (17)	
H6A	0.2024	0.6383	0.1751	0.070*	
H6B	0.2618	0.7307	0.1868	0.070*	
C7	0.5257 (10)	0.7313 (5)	0.1286 (10)	0.043 (3)	0.50
H7A	0.5182	0.7402	0.0632	0.052*	0.50
H7B	0.5951	0.7409	0.1775	0.052*	0.50
C8	0.4610 (5)	0.8015 (9)	0.1450 (8)	0.046 (3)	0.50
H8A	0.4716	0.7950	0.2117	0.056*	0.50
H8B	0.4787	0.8654	0.1371	0.056*	0.50
C9	0.2178 (4)	0.6113 (5)	−0.1472 (4)	0.0574 (17)	
H9A	0.2074	0.5823	−0.2083	0.069*	
H9B	0.1928	0.6755	−0.1617	0.069*	
C10	0.1636 (4)	0.5561 (5)	−0.1053 (4)	0.0563 (17)	
H10A	0.0925	0.5586	−0.1505	0.068*	
H10B	0.1844	0.4906	−0.0963	0.068*	
C1'	0.4867 (9)	0.7386 (5)	0.6036 (12)	0.058 (3)	0.50
H1'A	0.4973	0.7448	0.5473	0.070*	0.50
H1'B	0.5499	0.7505	0.6629	0.070*	0.50
C2'	0.4137 (5)	0.8140 (11)	0.5957 (12)	0.055 (4)	0.50
H2'A	0.4213	0.8262	0.6606	0.066*	0.50
H2'B	0.4268	0.8722	0.5713	0.066*	0.50
C7'	0.5261 (10)	0.7331 (5)	0.1712 (8)	0.043 (3)	0.50
H7'A	0.5935	0.7424	0.1839	0.052*	0.50
H7'B	0.5258	0.7431	0.2329	0.052*	0.50
C8'	0.4578 (5)	0.8049 (9)	0.0949 (10)	0.046 (3)	0.50
H8'A	0.4762	0.8684	0.1211	0.056*	0.50
H8'B	0.4628	0.8003	0.0352	0.056*	0.50

### Atomic displacement parameters (Å^2^)

**Table:**

	*U*^11^	*U*^22^	*U*^33^	*U*^12^	*U*^13^	*U*^23^
Zn1	0.0298 (3)	0.0409 (4)	0.0276 (3)	0.0003 (2)	0.0147 (3)	−0.0005 (2)
Zn2	0.0274 (3)	0.0432 (4)	0.0292 (3)	−0.0020 (2)	0.0144 (3)	0.0004 (2)
Cl1	0.0314 (7)	0.0538 (9)	0.0481 (8)	−0.0001 (6)	0.0161 (6)	0.0014 (7)
Cl2	0.0403 (8)	0.0667 (11)	0.0506 (9)	0.0023 (7)	0.0127 (7)	−0.0126 (8)
Cl3	0.0601 (9)	0.0463 (9)	0.0515 (9)	−0.0122 (7)	0.0330 (8)	−0.0077 (7)
Cl4	0.0544 (9)	0.0410 (8)	0.0751 (11)	−0.0058 (6)	0.0345 (8)	−0.0049 (8)
O1	0.159 (11)	0.238 (14)	0.172 (11)	0.013 (9)	0.136 (10)	−0.005 (9)
O2	0.095 (7)	0.076 (7)	0.060 (6)	0.013 (6)	−0.006 (5)	0.010 (5)
O3	0.128 (9)	0.074 (8)	0.158 (10)	−0.005 (7)	0.039 (8)	0.024 (7)
O4	0.049 (7)	0.066 (8)	0.059 (7)	0.008 (6)	0.012 (5)	0.012 (5)
O5	0.056 (6)	0.049 (6)	0.086 (8)	0.004 (5)	0.027 (5)	0.025 (5)
O6	0.160 (10)	0.167 (11)	0.134 (10)	0.010 (8)	0.088 (8)	−0.068 (8)
O7	0.106 (8)	0.069 (7)	0.116 (8)	−0.021 (6)	0.046 (6)	0.002 (6)
O8	0.041 (5)	0.054 (6)	0.058 (6)	−0.004 (4)	0.007 (4)	0.021 (5)
O9	0.059 (7)	0.084 (8)	0.060 (7)	−0.014 (6)	0.019 (5)	0.023 (6)
O10	0.056 (5)	0.066 (6)	0.101 (7)	0.002 (4)	0.043 (5)	−0.042 (5)
O11	0.108 (7)	0.095 (7)	0.063 (6)	−0.003 (6)	0.056 (6)	0.007 (5)
O12	0.044 (5)	0.051 (5)	0.070 (6)	−0.007 (4)	0.020 (5)	−0.034 (5)
O13	0.044 (5)	0.053 (6)	0.072 (6)	−0.020 (4)	0.017 (5)	−0.021 (5)
O14	0.081 (6)	0.080 (6)	0.083 (6)	−0.017 (5)	0.067 (5)	−0.012 (5)
O15	0.080 (7)	0.047 (5)	0.165 (10)	0.018 (5)	0.051 (6)	−0.001 (6)
O16	0.129 (8)	0.108 (8)	0.052 (5)	−0.039 (7)	0.032 (6)	0.008 (6)
O1'	0.054 (8)	0.067 (9)	0.079 (8)	0.006 (6)	0.034 (6)	−0.008 (6)
O2'	0.148 (10)	0.155 (10)	0.096 (8)	0.020 (8)	0.065 (7)	0.035 (7)
O3'	0.054 (5)	0.065 (6)	0.162 (9)	−0.007 (4)	0.068 (6)	−0.029 (6)
O4'	0.101 (7)	0.055 (6)	0.094 (7)	0.018 (5)	0.031 (6)	−0.020 (5)
O5'	0.062 (6)	0.079 (8)	0.086 (8)	−0.003 (5)	0.053 (6)	0.015 (6)
O6'	0.105 (7)	0.097 (7)	0.075 (6)	0.031 (6)	0.048 (5)	−0.020 (6)
O7'	0.117 (8)	0.063 (7)	0.113 (8)	0.003 (6)	0.026 (7)	−0.035 (6)
O8'	0.073 (7)	0.077 (8)	0.056 (7)	−0.028 (5)	0.013 (5)	0.027 (6)
O9'	0.053 (7)	0.091 (9)	0.067 (8)	−0.018 (6)	0.011 (5)	0.008 (6)
O10'	0.181 (13)	0.208 (14)	0.213 (14)	−0.029 (9)	0.088 (10)	0.059 (10)
O11'	0.176 (11)	0.146 (11)	0.125 (10)	−0.019 (8)	0.110 (9)	−0.022 (7)
O12'	0.119 (11)	0.101 (10)	0.148 (11)	−0.004 (7)	0.064 (8)	−0.039 (8)
O13'	0.111 (10)	0.082 (9)	0.168 (12)	−0.012 (7)	0.067 (8)	0.007 (7)
O14'	0.120 (9)	0.144 (11)	0.207 (12)	0.008 (8)	0.091 (9)	−0.003 (9)
O15'	0.207 (12)	0.197 (13)	0.152 (11)	−0.019 (9)	0.084 (9)	0.033 (9)
O16'	0.135 (9)	0.156 (10)	0.133 (9)	−0.029 (8)	0.089 (7)	−0.053 (8)
N1	0.033 (2)	0.059 (3)	0.038 (3)	0.008 (2)	0.016 (2)	0.002 (2)
N2	0.042 (3)	0.041 (3)	0.051 (3)	0.001 (2)	0.022 (2)	−0.001 (2)
N3	0.039 (2)	0.048 (3)	0.035 (2)	0.002 (2)	0.017 (2)	0.006 (2)
N4	0.034 (2)	0.066 (3)	0.033 (2)	−0.006 (2)	0.013 (2)	−0.001 (2)
N5	0.049 (3)	0.063 (3)	0.033 (2)	0.012 (2)	0.024 (2)	0.005 (2)
N6	0.051 (3)	0.094 (4)	0.040 (3)	0.018 (3)	0.029 (2)	0.020 (3)
N7	0.026 (2)	0.044 (3)	0.042 (2)	0.0059 (18)	0.0162 (19)	0.005 (2)
N8	0.033 (2)	0.045 (3)	0.051 (3)	0.006 (2)	0.019 (2)	0.008 (2)
N9	0.049 (3)	0.069 (4)	0.041 (3)	−0.001 (2)	0.028 (2)	−0.008 (3)
N10	0.036 (2)	0.064 (3)	0.042 (3)	−0.013 (2)	0.022 (2)	−0.011 (2)
C1	0.031 (4)	0.068 (6)	0.061 (9)	−0.010 (4)	0.012 (6)	−0.013 (6)
C2	0.050 (4)	0.038 (6)	0.082 (10)	−0.012 (4)	0.036 (5)	−0.031 (6)
C3	0.042 (3)	0.062 (4)	0.052 (3)	0.001 (3)	0.030 (3)	0.005 (3)
C4	0.035 (3)	0.051 (4)	0.047 (3)	−0.001 (2)	0.016 (3)	0.007 (3)
C5	0.093 (5)	0.055 (4)	0.061 (4)	0.018 (4)	0.056 (4)	0.019 (3)
C6	0.072 (5)	0.060 (4)	0.050 (4)	0.021 (3)	0.035 (3)	0.012 (3)
C7	0.029 (3)	0.052 (4)	0.048 (8)	0.000 (3)	0.019 (6)	0.014 (5)
C8	0.038 (4)	0.042 (4)	0.059 (7)	−0.007 (3)	0.024 (5)	−0.002 (6)
C9	0.059 (4)	0.071 (4)	0.031 (3)	−0.007 (3)	0.014 (3)	−0.004 (3)
C10	0.050 (4)	0.072 (5)	0.043 (3)	−0.015 (3)	0.021 (3)	−0.020 (3)
C1'	0.031 (4)	0.068 (6)	0.061 (9)	−0.010 (4)	0.012 (6)	−0.013 (6)
C2'	0.050 (4)	0.038 (6)	0.082 (10)	−0.012 (4)	0.036 (5)	−0.031 (6)
C7'	0.029 (3)	0.052 (4)	0.048 (8)	0.000 (3)	0.019 (6)	0.014 (5)
C8'	0.038 (4)	0.042 (4)	0.059 (7)	−0.007 (3)	0.024 (5)	−0.002 (6)

### Geometric parameters (Å, °)

**Table:**

Zn1—N2	2.108 (5)	N5—C5	1.455 (5)
Zn1—N5	2.111 (4)	N5—H51	0.8600
Zn1—N3	2.112 (4)	N5—H52	0.8600
Zn1—N1	2.148 (5)	N6—C6	1.459 (5)
Zn1—N4	2.185 (4)	N6—H61	0.8600
Zn2—N8	2.110 (5)	N6—H62	0.8600
Zn2—N10	2.129 (4)	N7—C7	1.465 (5)
Zn2—N7	2.135 (4)	N7—C7'	1.470 (5)
Zn2—N6	2.139 (5)	N7—H71	0.8600
Zn2—N9	2.147 (5)	N7—H72	0.8600
Cl1—O1	1.394 (5)	N7—H73	0.8600
Cl1—O3	1.397 (5)	N7—H74	0.8600
Cl1—O1'	1.403 (5)	N8—C8'	1.475 (5)
Cl1—O3'	1.404 (5)	N8—C8	1.477 (5)
Cl1—O2'	1.405 (5)	N8—H81	0.8600
Cl1—O4	1.415 (5)	N8—H82	0.8600
Cl1—O2	1.424 (5)	N8—H83	0.8600
Cl1—O4'	1.424 (5)	N8—H84	0.8600
Cl2—O7'	1.382 (5)	N9—C9	1.464 (5)
Cl2—O6	1.399 (5)	N9—H91	0.8600
Cl2—O8	1.401 (5)	N9—H92	0.8600
Cl2—O5	1.406 (5)	N10—C10	1.469 (4)
Cl2—O8'	1.407 (5)	N10—H101	0.8600
Cl2—O5'	1.408 (5)	N10—H102	0.8600
Cl2—O7	1.446 (5)	C1—C2	1.535 (6)
Cl2—O6'	1.452 (5)	C1—H1A	0.9800
Cl3—O9'	1.395 (5)	C1—H1B	0.9800
Cl3—O12	1.404 (4)	C2—H2A	0.9800
Cl3—O11'	1.407 (5)	C2—H2B	0.9800
Cl3—O12'	1.408 (5)	C3—C4	1.524 (5)
Cl3—O10	1.409 (4)	C3—H3A	0.9800
Cl3—O11	1.412 (5)	C3—H3B	0.9800
Cl3—O9	1.415 (5)	C4—H4A	0.9800
Cl3—O10'	1.422 (5)	C4—H4B	0.9800
Cl4—O16	1.402 (5)	C5—C6	1.518 (5)
Cl4—O14'	1.404 (5)	C5—H5A	0.9800
Cl4—O15	1.406 (5)	C5—H5B	0.9800
Cl4—O15'	1.407 (5)	C6—H6A	0.9800
Cl4—O13'	1.407 (5)	C6—H6B	0.9800
Cl4—O16'	1.411 (5)	C7—C8	1.536 (5)
Cl4—O14	1.415 (4)	C7—H7A	0.9800
Cl4—O13	1.415 (4)	C7—H7B	0.9800
N1—C1'	1.467 (5)	C8—H8A	0.9800
N1—C1	1.469 (5)	C8—H8B	0.9800
N1—H11	0.8600	C9—C10	1.524 (5)
N1—H12	0.8600	C9—H9A	0.9800
N1—H13	0.8600	C9—H9B	0.9800
N1—H14	0.8600	C10—H10A	0.9800
N2—C2'	1.466 (5)	C10—H10B	0.9800
N2—C2	1.467 (5)	C1'—C2'	1.530 (5)
N2—H21	0.8600	C1'—H1'A	0.9800
N2—H22	0.8600	C1'—H1'B	0.9800
N2—H23	0.8600	C2'—H2'A	0.9800
N2—H24	0.8600	C2'—H2'B	0.9800
N3—C3	1.467 (4)	C7'—C8'	1.537 (5)
N3—H31	0.8600	C7'—H7'A	0.9800
N3—H32	0.8600	C7'—H7'B	0.9800
N4—C4	1.469 (4)	C8'—H8'A	0.9800
N4—H41	0.8600	C8'—H8'B	0.9800
N4—H42	0.8600		
			
N2—Zn1—N5	106.3 (2)	C7—N7—Zn2	107.1 (6)
N2—Zn1—N3	103.55 (19)	C7'—N7—Zn2	105.8 (6)
N5—Zn1—N3	149.9 (2)	C7—N7—H71	110.3
N2—Zn1—N1	82.44 (17)	Zn2—N7—H71	110.3
N5—Zn1—N1	93.49 (19)	C7—N7—H72	110.3
N3—Zn1—N1	93.88 (17)	Zn2—N7—H72	110.3
N2—Zn1—N4	105.53 (18)	H71—N7—H72	108.5
N5—Zn1—N4	87.83 (19)	C7'—N7—H73	110.6
N3—Zn1—N4	80.81 (16)	Zn2—N7—H73	110.6
N1—Zn1—N4	171.23 (18)	C7'—N7—H74	110.6
N8—Zn2—N10	112.30 (19)	Zn2—N7—H74	110.6
N8—Zn2—N7	83.33 (15)	H73—N7—H74	108.7
N10—Zn2—N7	163.64 (19)	C8'—N8—Zn2	108.8 (6)
N8—Zn2—N6	99.7 (2)	C8—N8—Zn2	106.3 (6)
N10—Zn2—N6	89.49 (19)	C8—N8—H81	110.5
N7—Zn2—N6	92.61 (19)	Zn2—N8—H81	110.5
N8—Zn2—N9	99.1 (2)	C8—N8—H82	110.5
N10—Zn2—N9	80.59 (17)	Zn2—N8—H82	110.5
N7—Zn2—N9	92.53 (18)	H81—N8—H82	108.7
N6—Zn2—N9	160.9 (2)	C8'—N8—H83	109.9
O1—Cl1—O3	112.5 (7)	Zn2—N8—H83	109.9
O3—Cl1—O1'	108.4 (10)	C8'—N8—H84	109.9
O1'—Cl1—O3'	111.2 (6)	Zn2—N8—H84	109.9
O1'—Cl1—O2'	110.8 (7)	H83—N8—H84	108.3
O3'—Cl1—O2'	111.6 (6)	C9—N9—Zn2	108.3 (3)
O1—Cl1—O4	109.7 (7)	C9—N9—H91	110.0
O3—Cl1—O4	110.0 (6)	Zn2—N9—H91	110.0
O3'—Cl1—O4	106.8 (9)	C9—N9—H92	110.0
O1—Cl1—O2	108.5 (7)	Zn2—N9—H92	110.0
O3—Cl1—O2	107.9 (6)	H91—N9—H92	108.4
O4—Cl1—O2	108.0 (6)	C10—N10—Zn2	109.9 (3)
O1'—Cl1—O4'	110.8 (6)	C10—N10—H101	109.7
O3'—Cl1—O4'	105.3 (5)	Zn2—N10—H101	109.7
O2'—Cl1—O4'	107.0 (6)	C10—N10—H102	109.7
O4—Cl1—O4'	101.2 (8)	Zn2—N10—H102	109.7
O6—Cl2—O8	112.5 (7)	H101—N10—H102	108.2
O6—Cl2—O5	112.1 (6)	N1—C1—C2	102.9 (10)
O8—Cl2—O5	112.4 (6)	N1—C1—H1A	111.2
O7'—Cl2—O8'	113.3 (6)	C2—C1—H1A	111.2
O7'—Cl2—O5'	113.7 (6)	N1—C1—H1B	111.2
O8'—Cl2—O5'	109.8 (6)	C2—C1—H1B	111.2
O6—Cl2—O7	105.6 (6)	H1A—C1—H1B	109.1
O8—Cl2—O7	107.6 (6)	N2—C2—C1	108.4 (10)
O5—Cl2—O7	106.0 (6)	N2—C2—H2A	110.0
O7'—Cl2—O6'	107.4 (6)	C1—C2—H2A	110.0
O8'—Cl2—O6'	106.4 (6)	N2—C2—H2B	110.0
O5'—Cl2—O6'	105.7 (5)	C1—C2—H2B	110.0
O9'—Cl3—O11'	112.8 (7)	H2A—C2—H2B	108.4
O9'—Cl3—O12'	112.7 (7)	N3—C3—C4	108.7 (4)
O11'—Cl3—O12'	108.3 (7)	N3—C3—H3A	109.9
O12—Cl3—O10	109.5 (5)	C4—C3—H3A	109.9
O11'—Cl3—O10	70.1 (8)	N3—C3—H3B	109.9
O12'—Cl3—O10	130.2 (8)	C4—C3—H3B	109.9
O12—Cl3—O11	111.0 (6)	H3A—C3—H3B	108.3
O10—Cl3—O11	110.8 (5)	N4—C4—C3	107.8 (4)
O12—Cl3—O9	109.2 (6)	N4—C4—H4A	110.1
O10—Cl3—O9	108.4 (6)	C3—C4—H4A	110.1
O11—Cl3—O9	107.9 (6)	N4—C4—H4B	110.1
O9'—Cl3—O10'	108.8 (7)	C3—C4—H4B	110.1
O11'—Cl3—O10'	106.2 (7)	H4A—C4—H4B	108.5
O12'—Cl3—O10'	107.7 (7)	N5—C5—C6	119.4 (5)
O16—Cl4—O15	109.5 (6)	N5—C5—H5A	107.5
O14'—Cl4—O15'	109.9 (7)	C6—C5—H5A	107.5
O14'—Cl4—O13'	110.9 (7)	N5—C5—H5B	107.5
O15'—Cl4—O13'	110.9 (7)	C6—C5—H5B	107.5
O14'—Cl4—O16'	108.8 (6)	H5A—C5—H5B	107.0
O15'—Cl4—O16'	107.0 (7)	N6—C6—C5	120.1 (5)
O13'—Cl4—O16'	109.3 (7)	N6—C6—H6A	107.3
O16—Cl4—O14	108.9 (6)	C5—C6—H6A	107.3
O15—Cl4—O14	109.2 (6)	N6—C6—H6B	107.3
O16—Cl4—O13	112.5 (6)	C5—C6—H6B	107.3
O15—Cl4—O13	109.5 (5)	H6A—C6—H6B	106.9
O16'—Cl4—O13	119.2 (8)	N7—C7—C8	108.3 (9)
O14—Cl4—O13	107.2 (5)	N7—C7—H7A	110.0
C1'—N1—Zn1	106.3 (6)	C8—C7—H7A	110.0
C1—N1—Zn1	106.6 (6)	N7—C7—H7B	110.0
C1—N1—H11	110.4	C8—C7—H7B	110.0
Zn1—N1—H11	110.4	H7A—C7—H7B	108.4
C1—N1—H12	110.4	N8—C8—C7	109.3 (9)
Zn1—N1—H12	110.4	N8—C8—H8A	109.8
H11—N1—H12	108.6	C7—C8—H8A	109.8
C1'—N1—H13	110.5	N8—C8—H8B	109.8
Zn1—N1—H13	110.5	C7—C8—H8B	109.8
C1'—N1—H14	110.5	H8A—C8—H8B	108.3
Zn1—N1—H14	110.5	N9—C9—C10	108.2 (5)
H13—N1—H14	108.7	N9—C9—H9A	110.0
C2—N2—Zn1	103.4 (7)	C10—C9—H9A	110.0
C2—N2—H21	111.1	N9—C9—H9B	110.0
Zn1—N2—H21	111.1	C10—C9—H9B	110.0
C2—N2—H22	111.1	H9A—C9—H9B	108.4
Zn1—N2—H22	111.1	N10—C10—C9	108.2 (5)
H21—N2—H22	109.0	N10—C10—H10A	110.1
C2'—N2—H23	108.9	C9—C10—H10A	110.1
Zn1—N2—H23	108.9	N10—C10—H10B	110.1
C2'—N2—H24	108.9	C9—C10—H10B	110.1
Zn1—N2—H24	108.9	H10A—C10—H10B	108.4
H23—N2—H24	107.7	N1—C1'—C2'	115.6 (11)
C3—N3—Zn1	108.6 (3)	N1—C1'—H1'A	108.4
C3—N3—H31	110.0	C2'—C1'—H1'A	108.4
Zn1—N3—H31	110.0	N1—C1'—H1'B	108.4
C3—N3—H32	110.0	C2'—C1'—H1'B	108.4
Zn1—N3—H32	110.0	H1'A—C1'—H1'B	107.4
H31—N3—H32	108.4	N2—C2'—C1'	109.2 (9)
C4—N4—Zn1	108.0 (3)	N2—C2'—H2'A	109.8
C4—N4—H41	110.1	C1'—C2'—H2'A	109.8
Zn1—N4—H41	110.1	N2—C2'—H2'B	109.8
C4—N4—H42	110.1	C1'—C2'—H2'B	109.8
Zn1—N4—H42	110.1	H2'A—C2'—H2'B	108.3
H41—N4—H42	108.4	N7—C7'—C8'	111.6 (9)
C5—N5—Zn1	111.4 (3)	N7—C7'—H7'A	109.3
C5—N5—H51	109.4	C8'—C7'—H7'A	109.3
Zn1—N5—H51	109.4	N7—C7'—H7'B	109.3
C5—N5—H52	109.4	C8'—C7'—H7'B	109.3
Zn1—N5—H52	109.4	H7'A—C7'—H7'B	108.0
H51—N5—H52	108.0	N8—C8'—C7'	107.2 (9)
C6—N6—Zn2	115.7 (4)	N8—C8'—H8'A	110.3
C6—N6—H61	108.4	C7'—C8'—H8'A	110.3
Zn2—N6—H61	108.4	N8—C8'—H8'B	110.3
C6—N6—H62	108.4	C7'—C8'—H8'B	110.3
Zn2—N6—H62	108.4	H8'A—C8'—H8'B	108.5
H61—N6—H62	107.4		
			
N2—Zn1—N1—C1'	−18.1 (7)	N10—Zn2—N8—C8	168.6 (6)
N5—Zn1—N1—C1'	87.9 (7)	N7—Zn2—N8—C8	−16.4 (6)
N3—Zn1—N1—C1'	−121.3 (7)	N6—Zn2—N8—C8	75.1 (6)
N2—Zn1—N1—C1	15.8 (7)	N9—Zn2—N8—C8	−107.9 (6)
N5—Zn1—N1—C1	121.8 (7)	N8—Zn2—N9—C9	−93.8 (4)
N3—Zn1—N1—C1	−87.4 (7)	N10—Zn2—N9—C9	17.4 (4)
N5—Zn1—N2—C2'	−93.8 (9)	N7—Zn2—N9—C9	−177.5 (4)
N3—Zn1—N2—C2'	89.9 (9)	N6—Zn2—N9—C9	77.0 (7)
N1—Zn1—N2—C2'	−2.3 (9)	N8—Zn2—N10—C10	108.6 (4)
N4—Zn1—N2—C2'	173.9 (8)	N7—Zn2—N10—C10	−53.6 (8)
N5—Zn1—N2—C2	−72.2 (6)	N6—Zn2—N10—C10	−151.1 (4)
N3—Zn1—N2—C2	111.4 (6)	N9—Zn2—N10—C10	12.5 (4)
N1—Zn1—N2—C2	19.3 (6)	C1'—N1—C1—C2	49.1 (13)
N4—Zn1—N2—C2	−164.5 (6)	Zn1—N1—C1—C2	−45.4 (10)
N2—Zn1—N3—C3	86.0 (4)	C2'—N2—C2—C1	68 (3)
N5—Zn1—N3—C3	−87.1 (5)	Zn1—N2—C2—C1	−51.6 (10)
N1—Zn1—N3—C3	169.1 (4)	N1—C1—C2—N2	67.4 (12)
N4—Zn1—N3—C3	−17.9 (4)	Zn1—N3—C3—C4	45.0 (5)
N2—Zn1—N4—C4	−114.1 (4)	Zn1—N4—C4—C3	39.4 (5)
N5—Zn1—N4—C4	139.6 (4)	N3—C3—C4—N4	−57.2 (6)
N3—Zn1—N4—C4	−12.5 (4)	Zn1—N5—C5—C6	−169.5 (5)
N2—Zn1—N5—C5	−28.7 (5)	Zn2—N6—C6—C5	−161.0 (5)
N3—Zn1—N5—C5	144.3 (4)	N5—C5—C6—N6	−41.0 (10)
N1—Zn1—N5—C5	−111.8 (5)	C7'—N7—C7—C8	−51.0 (16)
N4—Zn1—N5—C5	76.9 (5)	Zn2—N7—C7—C8	40.2 (10)
N8—Zn2—N6—C6	56.0 (5)	C8'—N8—C8—C7	−56.4 (14)
N10—Zn2—N6—C6	−56.6 (5)	Zn2—N8—C8—C7	43.1 (10)
N7—Zn2—N6—C6	139.6 (5)	N7—C7—C8—N8	−57.5 (13)
N9—Zn2—N6—C6	−114.9 (6)	Zn2—N9—C9—C10	−43.4 (6)
N8—Zn2—N7—C7	−13.6 (6)	Zn2—N10—C10—C9	−39.2 (6)
N10—Zn2—N7—C7	149.8 (7)	N9—C9—C10—N10	55.6 (7)
N6—Zn2—N7—C7	−113.1 (6)	C1—N1—C1'—C2'	−58.6 (18)
N9—Zn2—N7—C7	85.3 (6)	Zn1—N1—C1'—C2'	36.8 (14)
N8—Zn2—N7—C7'	13.8 (6)	C2—N2—C2'—C1'	−46 (2)
N10—Zn2—N7—C7'	177.2 (7)	Zn1—N2—C2'—C1'	21.6 (16)
N6—Zn2—N7—C7'	−85.7 (6)	N1—C1'—C2'—N2	−39.9 (19)
N9—Zn2—N7—C7'	112.7 (6)	C7—N7—C7'—C8'	56.5 (16)
N10—Zn2—N8—C8'	−160.0 (6)	Zn2—N7—C7'—C8'	−40.3 (10)
N7—Zn2—N8—C8'	15.0 (6)	C8—N8—C8'—C7'	50.7 (13)
N6—Zn2—N8—C8'	106.5 (6)	Zn2—N8—C8'—C7'	−39.5 (11)
N9—Zn2—N8—C8'	−76.5 (6)	N7—C7'—C8'—N8	55.0 (13)

### Hydrogen-bond geometry (Å, °)

**Table:**

*D*—H···*A*	*D*—H	H···*A*	*D*···*A*	*D*—H···*A*
N1—H12···O1	0.86	1.93	2.790 (9)	174
N1—H13···O8'	0.86	2.22	3.066 (12)	169
N3—H32···O12	0.86	2.34	3.018 (7)	136
N3—H32···O12'	0.86	2.29	3.070 (10)	152
N4—H41···O7^i^	0.86	2.35	3.157 (12)	156
N4—H41···O7'^i^	0.86	2.08	2.885 (9)	156
N4—H42···O16'	0.86	2.12	2.973 (8)	175
N5—H51···O1	0.86	2.30	3.120 (15)	159
N6—H62···O2	0.86	2.29	3.034 (11)	146
N7—H71···O2	0.86	2.32	3.163 (10)	168
N7—H71···O2'	0.86	2.22	3.018 (10)	154
N7—H74···O6^ii^	0.86	2.35	3.143 (11)	154
N7—H74···O6'^ii^	0.86	2.36	3.210 (9)	172
N8—H81···O11^ii^	0.86	2.26	3.075 (8)	157
N8—H81···O11'^ii^	0.86	2.29	3.148 (12)	177
N10—H102···O3^i^	0.86	2.17	2.929 (9)	146

Symmetry codes: (i) *x*−1/2, −*y*+3/2, *z*−1/2; (ii) *x*, *y*, *z*−1.
